# Microbiomes and Resistomes in Biopsy Tissue and Intestinal Lavage Fluid of Colorectal Cancer

**DOI:** 10.3389/fcell.2021.736994

**Published:** 2021-09-17

**Authors:** Yumeng Yuan, Yihuan Chen, Fen Yao, Mi Zeng, Qingdong Xie, Muhammad Shafiq, Sohail Muhammad Noman, Xiaoyang Jiao

**Affiliations:** ^1^Department of Cell Biology and Genetics, Shantou University Medical College, Shantou, China; ^2^The Second Affiliated Hospital of Shantou University Medical College, Shantou, China; ^3^Guangdong Provincial Key Laboratory of Infectious Diseases and Molecular Immunopathology, Shantou, China; ^4^Department of Pharmacology, Shantou University Medical College, Shantou, China

**Keywords:** colorectal cancer, intestinal lavage fluid, gut microbiome, resistome, bacterial cluster

## Abstract

**Aim:** The gut microbiome plays a crucial role in colorectal cancer (CRC) tumorigenesis, but compositions of microorganisms have been inconsistent in previous studies due to the different types of specimens. We investigated the microbiomes and resistomes of CRC patients with colonic biopsy tissue and intestinal lavage fluid (IVF).

**Methods:** Paired samples (biopsy tissue and IVF) were collected from 20 patients with CRC, and their gut microbiomes and resistomes were measured by shotgun metagenomics. Clinical and laboratory data were recorded. Bioinformatics (KneadData, Kraken2, and FMAP) and statistical analysis were done using the R (v4.0.2) software.

**Results:** Bacterial diversity in IVF was higher than in tissue samples, and bacterial operational taxonomic units (OTUs) were 2,757 in IVF vs. 197 in tissue. β-diversity showed distinct clusters in paired samples. The predominant bacteria in IVF were phylum Proteobacteria, while the predominant bacteria of tissue were phylum Actinobacteria. Twenty-seven representative bacteria were selected to form six bacterial clusters, which showed only Firmicutes Cluster 1, and the Bacteroidetes Cluster 1 were significantly more abundant in the IVF group than those in the tissue group (*p* < 0.05). The Firmicutes Cluster 2, Bacteroidetes Cluster 2, Pathogen Cluster, and *Prevotella* Cluster were not significantly different between IVF and tissue (*p* > 0.05). Correlation analysis revealed that some bacteria could have effects on metabolic and inflammatory parameters of CRC patients. A total of 1,295 antibiotic resistance genes (ARGs) were detected in the gut microbiomes, which conferred multidrug resistance, as well as resistance to tetracycline, aminoglycoside, and more. Co-occurrence patterns revealed by the network showed mainly ARG-carrying bacteria to be similar between IVF and tissue, but leading bacteria located in the hub differed between IVF and tissue.

**Conclusion:** Heterogeneity of microbiota is particularly evident when studied with IVF and tissue samples, but bacterial clusters that have close relationships with CRC carcinogenesis are not significantly different, using IVF as an alternative to tissue for gut microbiome, and resistome assessment may be a feasible method.

## Introduction

Colorectal cancer (CRC) represents approximately 10% of the global cancer incidence, and also is the third most diagnosed cancer (10.2% of total cases) and the second leading cause of cancer death (9.2% of total cases) worldwide (Vogelstein et al., 2013; Bray et al., 2018). Despite extensive effort, the carcinogenesis of CRC is still not fully understood. Currently, the role of the intestinal microbiome in the pathogenesis of CRC has become increasingly important. The gut microbiota or its metabolites is a key environmental factor influencing colon tumorigenesis, which is usually associated with altered microbial diversity, increased abundance of pathogenic microbes, or depletion of protective microbes (Wong and Yu, 2019). The intimate crosstalk between the gut microbial community and the epithelium layer of the host is a critical factor for cell proliferation and differentiation, gene expression in host epithelial cells, and regulation of inflammation (Hasegawa et al., 2007; Arthur et al., 2014). Recent studies revealed that various bacteria, including *Fusobacterium* sp. (*Fn*), *Escherichia coli*, *Enterococcus faecalis*, *Streptococcus gallolyticus*, and *enterotoxigenic Bacteroides fragilis*, are microorganisms that are closely associated with CRC carcinogenesis (Yoon et al., 2017). Therefore, characterization of the tumor microbiome is an essential step in unraveling the effects of bacteria on cancer hallmarks (Nejman et al., 2020).

Presently, the role of microorganisms in CRC carcinogenesis has not been unified nor conclusive (Flemer et al., 2017). Because of the complex network and interaction between microorganisms, intestinal microbiota may have a greater ability to influence the intestinal microenvironment than a single bacterium. Therefore, a study on intestinal microbiota may provide more information on CRC carcinogenesis. However, current studies in understanding intestinal microbiota complexity and dynamics are inconsistent (Kostic et al., 2012; Zeller et al., 2014; Flemer et al., 2017). The variability may be due to different detection methodologies, tumor location, or sampling stages. There are currently three main types of samples for intestinal microbiome detection: feces, mucosal tissue, and intestinal lavage samples. As feces samples are being acquired relatively easily, most studies assess gut microbial diversity through analysis of fecal samples. However, feces, reflecting the fecal–luminal microbiota, are largely influenced and continually changed by various factors, including diet, time of sampling, and antibiotic use. This instability largely affects their accuracy in mirroring the microbial structure. Moreover, microbial diversity at the mucosal surface is hard to reflect using fecal samples (Zoetendal et al., 2002; Eckburg et al., 2005). Studies have confirmed significant differences existing in microbial structure and community composition between normal fecal and mucosal samples and found fecal microbiota to be less representative of disease-associated dysbiosis than their mucosal counterparts, especially among CRC patients (Zoetendal et al., 2002; Eckburg et al., 2005; Chen et al., 2012). Mucosal biopsies display greater microbial diversity, taxonomic and phylogenetic differences (Durban et al., 2011; Li et al., 2015), and contrasting dominant bacterial populations (Zoetendal et al., 2002). However, obtaining a mucosa sample requires bowel preparation and biopsy. The procedure is invasive and has difficultly being accepted by the participants. Thus, the clinical applicability of biopsy tissue as a screening method for CRC is significantly reduced. In addition, the spatial organization of bacteria along the gastrointestinal tract is highly variable (Donaldson et al., 2016), causing microbial diversity to depend, to a large extent, on the anatomical site sampled rather than the entire intestine. Thus, considerable cohort-to-cohort differences have been reported among mucosal microbial taxa from CRC patients (Zeller et al., 2014; Burns et al., 2015; Nakatsu et al., 2015). Bowel preparation before biopsy may change the microbiota composition. Decreases in richness and microbial structure similarity after extensive colonic lavage have also been observed (Harrell et al., 2012). Intestinal lavage fluid (IVF) is obtained from patients preparing for laparoscopic colorectal resection and is very easy to aspirate, through a suction channel in the colonoscope, directly into a collecting tube. Some species of mucosa-associated microbiota (either on the surface or in cavities) may be obtained after a few intestinal rinses (Shen et al., 2020). A previous study showed that IVF contains higher microbial counts than corresponding biopsy samples, suggesting that IVF better reflects the microbial composition of a mucosal biopsy (Watt et al., 2016). Although detection of microbiota in IVF has several advantages in mirroring mucosa-associated microbiota (Shen et al., 2020), data correlating tumor tissue with microbiota from IVF are sparse. In this study, both tumor tissue and IVF samples were collected simultaneously, and multifaceted comparisons were made between the two sample types.

## Materials and Methods

### Patients

The Shantou University Medical College Institutional Ethics Board approved the study including all procedures (participant recruitment and all experimental protocols). Patients who were selected from the Second Affiliated Hospital of Shantou University provided written informed consent. Pathological tests diagnosed 20 CRC patients. Patients with a history of polyps, adenomas, or non-primary CRC were excluded. None of the patients received antibiotics before sample collection. All participants were Chinese living in Guangdong Province, China. No antibiotics or prebiotics were used in these patients before the sample collection.

### Sample Collection

All participants underwent a similar bowel cleansing procedure. For IVF collection, the patient fasted for 1 day before sample collection. Then, all patients were given a 500-ml enema, and all liquid discharged from the intestine was collected. Samples were centrifuged (4°C, 10,000 × *g*, 10 min) within 2 h. The sediments were collected and stored at −80°C. Bowel preparation was good in all subjects in the study. Tissue samples [biopsy (Bx)] were collected from 20 participants after CRC screening colonoscopy. Subsequent histological analysis was performed on all participants. Paired samples [biopsy (Bx)] and IVF were collected from the 20 CRC participants.

### Shotgun Metagenomic Detection for Tumor Tissue and Intestinal Lavage Fluid

Bacterial genetic DNA was isolated from tissue/IVF samples using an AllPrep DNA/RNA kit (Qiagen, German). A DNA quality test was performed by using Qubit dsDNA Assay KitinQubit 2.0 Fluorometer (Life Technologies, CA, United States), and then the qualified DNA samples with an OD value between 1.8 and 2.0 were accepted, and about 1 μg of DNA from each sample was used to construct a library. Sequencing libraries were generated using NEB NextUltra DNA Library Prep Kit for Illumina (NEB, United States). PCR products were purified (AMPure XP system), and libraries were analyzed for size distribution by Agilent2100 Bioanalyzer and quantified using qPCR. Water was used as the negative control. The clustering of the index-coded samples was performed on a cBot Cluster Generation System. DNA samples was used for shotgun library construction. Subsequently, Illumina high-throughput sequencing was performed with the NovaSeq 6000 platform (paired-end sequencing, PE150 × 2). The bacterial genomic sequences identified in this study were deposited in the NCBI Sequence Read Archive with accession numbers (PRJNA754518), which can be shared with readers.

### Bioinformatics Analysis Workflow

For subsequent bioinformatics analysis, the KneadData software was used for quality control of raw data (based on Trimmomatic) and de-hosting (based on Bowtie2). Before and after KneadData, FastQC was used to detect the rationality and effect of quality control, and tags were clustered to OTU at 97% sequence similarity. Taxonomic ranks were assigned to the OTU representative sequence using the Kraken2. The diversity indices were calculated by the R software (v4.0.2). Alpha diversity, beta diversity, and the different species screening were analyzed based on OTU and taxonomic ranks. Starting from clean reads with host genes removed, FMAP software was used to compare and annotate the reads of each sample with the antibiotic resistance gene database (CARD) to check for the ARGs conferring resistance to aminoglycoside, tetracycline, beta-lactam, colistin, fosfomycin, fusidic acid, macrolide, nitroimidazole, oxazolidinone, phenicol, quinolone, rifampicin, sulfonamide, trimethoprim, and glycopeptide antibiotics.

### Statistical Analyses

Mann–Whitney U (non-parametric) and Dunn’s tests were employed to analyze the differences in the abundance between two groups for non-normally distributed data. Data were analyzed using the R software (v4.0.2), SPSS 23.0 software, and *p*-values represent two-sided statistical tests. All graphics were made with GraphPad Prism (v 8.0.2) and R (v4.0.2). Spearman’s correlation analysis was employed to assess associations between ARG subtypes and bacterial species. Pairs with thresholds of correlation coefficient >0.4, *p* < 0.05, and the two items occurring in more than half of the samples were selected to build the network using the software Gephi (v 0.9.2).

Co-occurring network analysis using the Spearman rank correlation was conducted using Hmisc (Harrell, Vanderbilt University School of Medicine, Nashville, TN, United States) within the R software package, using the relative abundance of different types of bacterial genera. Each co-occurring pair had an absolute Spearman rank correlation above 0.4, with a significance level under 0.05. The results were transformed into links between two bacterial taxa in the co-occurrence network. Co-occurring networks were visualized using Gephi (v 0.9.2). Spearman rank correlation analysis between ARGs and bacterial communities was performed in R with the psych package. Univariate analysis using a Mann–Whitney U-test was performed to assess associations between ARGs and gut microbiota and clinical factors.

## Results

### Clinical and Demographic Information of Patients

Twenty newly diagnosed CRC patients were enrolled in our study, including 10 males and 10 females, and their average age was 58.40 ± 2.36 years. Tumors of four (20%) patients were in the proximal colon, tumors of eight (40%) patients were located in the distal colon, and tumors of eight (40%) patients were in the rectum. Fourteen (70%) patients were in I–II stage of tumor node metastasis (TNM I–II), and six (30%) patients were in TNM III–IV. Seven (35%) patients had hypertension. The tumor marker CA-19-9 was significantly higher in CRC patients (155.09 ± 585.59; reference range 0–37 U/ml). Other tumor markers and biochemistry parameters in CRC patients were not significantly different compared with reference range (Table 1).

**TABLE 1:** Clinical characteristics of participants.

**Indexes**	**Tumor patients**	**Reference range**
Age (years)	58.40 ± 2.36	
**Gender**		
Female	10 (50%)	
Male	10 (50%)	
**Location of tumor**		
Proximal colon	4(20%)	
Distal colon	8 (40%)	
Rectum	8 (40%)	
**TNM**		
0–II	14 (70%)	
III–IV	6 (30%)	
**Blood pressure**		
Hypertension	7 (35%)	
Normotension	13 (65%)	
**LNM**		
Yes	6 (30%)	
No	14 (70%)	
**BMI,** kg/m^2^	22.68 ± 3.53	
**Laboratory parameters (M ± Q)**		
Glu, mmol/L	6.14 ± 1.50	3.90–6.10
T-CH, mmol/L	4.56 ± 0.63	3.10–5.71
TG, mmol/L	1.81 ± 0.35	0.58–1.70
HDL, mmol/L	1.25 ± 0.26	0.91–1.55
LDL, mmol/L	2.77 ± 0.47	2.07–3.12
CEA, μg/L	4.39 ± 5.97	0–5
CA-19-9, U/ml	155.09 ± 585.59	0–37
TBA, μmol/L	4.78 ± 4.87	0–10
LDH, U/L	180.30 ± 46.00	120.0–250.0
ADA, U/L	10.05 ± 3.08	0–25
NEUT#, 10^9^/L	4.40 ± 1.49	1.8–6.3
LY#, 10^9^/L	1.52 ± 0.44	1.1–3.2
PLT, 10^9^/L	275.45 ± 78.40	125–350
NLR	3.35 ± 2.28	
PLR	199.43 ± 90.07	

*TNM, tumor–node–metastasis; LNM, lymph node metastasis; BMI, body mass index; Glu, glucose; T-CH, total cholesterol; TG, triglyceride; HDL, high-density lipoprotein cholesterol; LDL, low-density lipoprotein cholesterol; CEA, carcinoembryonic antigen; CA199, carbohydrate antigen 19-9; TBA, total bile acid; LDH, lactate dehydrogenase; ADA, adenosine deaminase; NEUT#, neutrophils; LY#, lymphocyte; PLT, platelet count; NLR, neutrophil–lymphocyte ratio; PRL, platelet-to-lymphocyte ratio.*

### Comparison of Gut Bacterial Communities Between Intestinal Lavage Fluid and Tissue Samples

In this study, we analyzed a total of 20 paired tissue and IVF samples, with each pair being taken from one subject. After filtering raw data with the criteria, we obtained a dataset consisting of 350,606,294 high-quality gene sequence reads, with an average of 8,765,157 (*n* = 40) sequences per sample. Within the dataset, we identified a total of 2,758 OTUs, based on 97% sequence similarity (equal to bacterial species level), with an average of 323.35 ± 386.37(*n* = 40) OTUs per sample.

### Diversity and Taxonomical Measurements of the Microbiome Data

**FIGURE 1:**
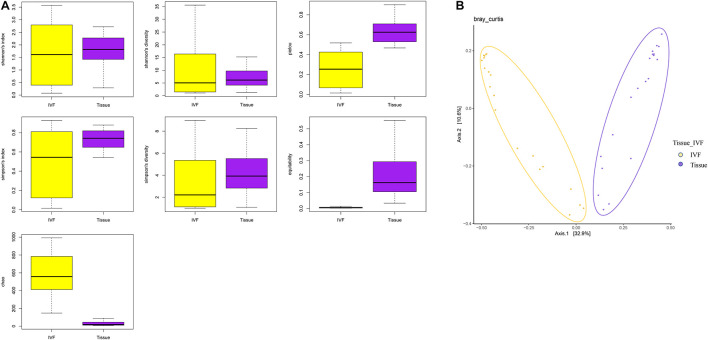
**(A)** Boxplots represent the indices of observed species. Chao, equitability, Pielou, Shannon’s diversity, and Simpson’s diversity indices in the IVF and tissue groups. IVF, intestinal lavage fluid; tissue, tumor tissue. **(B)** Beta diversity between the two groups. PCoA analyses. The abscissa is a principal component, the ordinate is another principal component, and the percentage on the coordinate axis indicates the contribution of the two principal components to the sample difference.

Comparisons of alpha and beta diversity were performed between the two types of samples. Alpha diversity includes Observed species, Chao, Ace, Shannon, and Simpson indices. Shannon’s diversity and index were similar between the two groups, while Simpson’s diversity and index, although insignificant, tended to be higher in the tissue group than in the IVF group (*p* > 0.05). The higher the Simpson index, the lower the bacterial diversity. Our results suggested that the gut microbiome had higher bacterial diversity in IVF than in tissue samples. The Chao index (species richness) was higher in IVF than in tissue (*p* < 0.05), while equitability and Pielou evenness were significantly lower in IVF than in tissue (*p* < 0.05) (617.35, 0.006, and 0.243 in IVF vs. 29.35, 0.236, and 0.596 in the tissue group, respectively), and Good’s coverage demonstrated no statistical differences between the two groups (Figure 1A). Beta diversity was analyzed by the QIIME software (v1.80) to reveal the differences in species complexity. PCoA performed on the Bray–Curtis distance index, measured in unannotated OTUs, revealed distinguishing bacterial species distributions between tissue and normal IVF groups. Our results showed that microflora clustered strongly by samples. Paired samples (tissue vs. IVF) of the 20 subjects formed distinct clusters at the family level. Alpha and beta diversities in the two sample groups are shown in Figure 1B.

### Comparison of Bacteria at the Phyla, Genus, and Species Levels Between Intestinal Lavage Fluid and Tissue

This study also evaluated the similarity (or dissimilarity) of taxa in IVF and tissue samples. We performed taxonomical analysis on the 2,757 OTUs in IVF and 197 OTUs in tissue, excluding the unclassified OTUs. Among the classified OTUs, we identified 19 phyla, 42 classes, 100 orders, 221 families, 859 genera, and 2,747 species in IVF, in contrast to the 11 phyla, 21 classes, 33 orders, 52 families, 102 genera, and 197 species in the tissue group.

**FIGURE 2:**
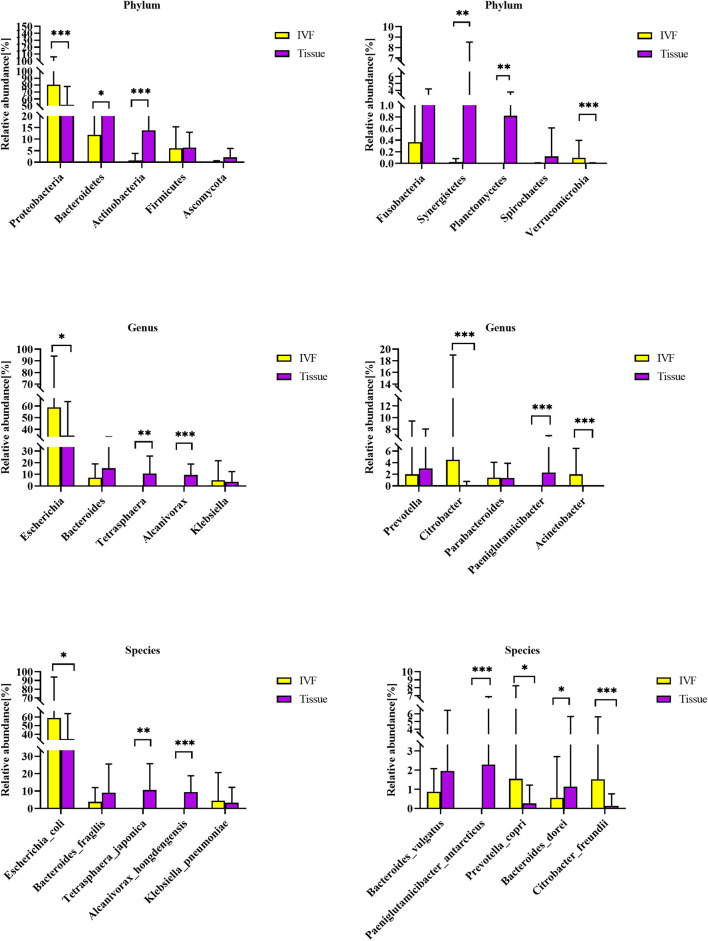
Histogram of IVF and tissue at the phylum, genus, and species levels. IVF, intestinal lavage fluid; tissue, tumor tissue. ^∗∗∗^*p* < 0.001, ^∗∗^*p* < 0.01, ^∗^*p* < 0.05.

First, we studied the differences between the two groups of samples at the top 10 phyla level by Dunn’s test. We identified six phyla that were significantly different between the two groups. Phyla Proteobacteria and Verrucomicrobia were higher in IVF, while Bacteroidetes, Actinobacteria, Synergistetes, and Planctomycetes were higher in tumor tissue (*p* < 0.05). Fusobacteria tended to be higher in tissue than in IVF (*p* > 0.05). In the top 10 genera, *Escherichia*, *Citrobacter*, and *Acinetobacter* were higher in IVF, and *Tetrasphaera*, *Alcanivorax*, and *Paeniglutamicibacter* were higher in tumor tissue. In the top 10 bacteria species detected, there were seven species that were significantly different between the two groups. *Escherichia coli*, *Prevotella copri*, and *Citrobacter freundii* were higher in IVF, while *Tetrasphaera japonica*, *Alcanivorax hongdengensis*, *Paeniglutamicibacter antarcticus*, and *Bacteroides dorei* were higher in tissue. *Klebsiella pneumoniae* was insignificantly higher in IVF, and *Bacteroides fragilis* and *Bacteroides vulgatus* were insignificantly higher in tissue. Comparisons between the two samples of the top 10 bacterial phyla, genera, and species are shown in Figure 2.

### Bacterial Co-abundance Groups in Intestinal Lavage Fluid and Tissue of Colorectal Cancer Patients

**FIGURE 3:**
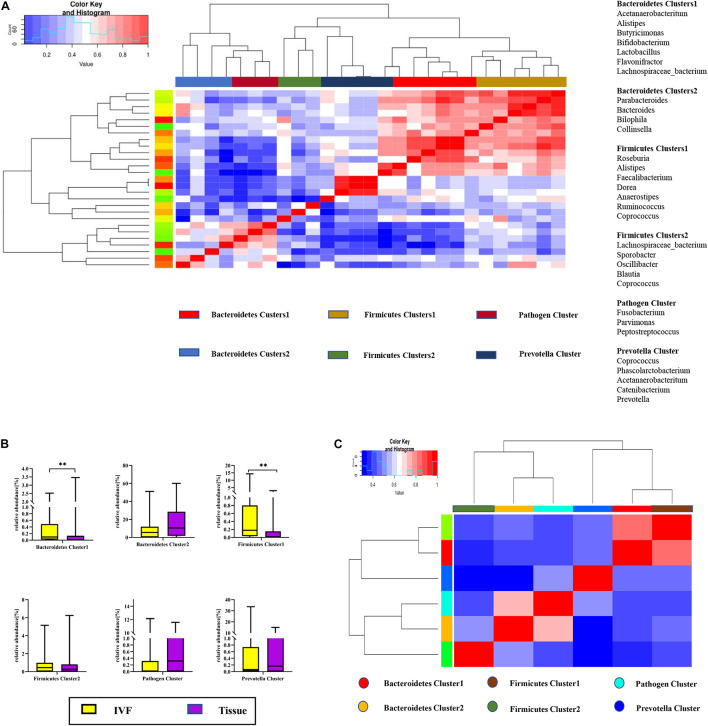
**(A)** Hierarchical Ward-linkage clustering based on the Pearson correlation coefficients of the relative abundance of operational taxonomic units in IVF and tissue microbiota of 20 individuals with colorectal cancer (CRC). Bacterial clusters were defined based on the clusters in the vertical tree and named after their most notable characteristic. Column color coding is according to the legend below. The right shows the most abundant bacterial genera, and the most strongly connected genera in each bacterial cluster (i.e., genera with the highest numbers of significant positive correlations with other members of each respective group) are listed. **(B)** Boxplots of the relative abundances of the six co-abundance groups (CAGs) of bacterial clusters, **p < 0.01. **(C)** Hierarchical Ward-linkage clustering based on the Pearson correlation coefficients of the relative abundance of CAGs in CRC samples (20 individuals).

Co-abundance groups (CAGs) resemble the previously formulated concept of enterotypes, which reflect community structure that could be more informative than abundance differences of individual taxa (Claesson et al., 2012; Flemer et al., 2017). According to previous studies and the literature analyses, we selected 27 representative bacteria that play crucial roles in CRC pathogenesis to determine which bacterial clusters were the primary drivers of the microbiome (either in IVF or in tissue samples). First, the Spearman correlation coefficient was calculated, and according to the correlation coefficient, the R language was used for cluster heatmap analysis. Six robust CAGs or bacterial clusters in the OTU dataset were identified, including Bacteroidetes1, Bacteroidetes 2, Firmicutes1, Firmicutes 2, Pathogen Cluster, and *Prevotella* Cluster (Flemer et al., 2017) (Figure 3A).

Then, the abundance of each cluster was compared between the two groups. Our results showed that Firmicutes Cluster 1 and Bacteroidetes Cluster 1 were significantly more abundant in the IVF group than those in the tissue group (*p* < 0.05), Firmicutes Cluster 2 tended to be more abundant in the IVF group than those in the tissue group, but the difference was insignificant. Conversely, Bacteroidetes 2, Pathogen Cluster, and *Prevotella* Cluster tended to be less abundant in the IVF group than those in the tissue group, but the difference was not statistically significant (*p* > 0.05) (Figures 3B,C).

**FIGURE 4:**
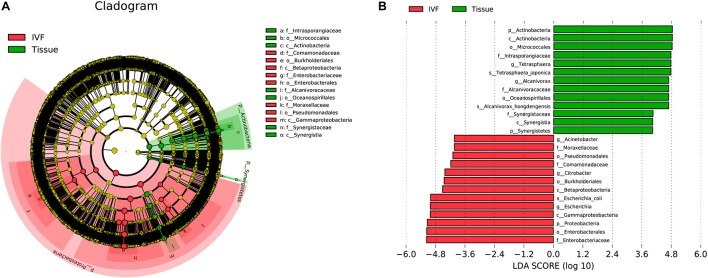
System clustering tree. **(A)** The red color indicates IVF, the green color represents the tissue. The red nodes represent the key species in the IVF group; the green nodes represent the key species in the tissue group. The yellow nodes represent normal species. The species’ name is on the right side of the figure. **(B)** The key species in the two groups are analyzed by linear discriminant analysis (LDA) analysis. The LDA score is represented.

In order to determine which bacterial taxa are the main drivers of IVF and tissue microbiota, linear discriminant analysis (LDA) was used to calculate the effect size of different bacterial taxa. An LDA > 2 indicates significant differences among species, and the larger the LDA, the greater the species difference. In the phylogenetic tree, the circle radiating from the inside to the outside represents the classification level from the phylum to the genus. Each node represents a species, and the diameter of each small circle was proportional to the relative abundance of the taxon. The yellow nodes indicate that the differences among the two groups were not significant, the red nodules represent the main bacteria that play an important role in IVF, and the green nodules represent the bacteria that play a crucial role in tissue. The dominant bacteria in IVF (LDA > 4) were Proteobacteria (phylum level), mainly Betaproteobacteria and Gammaproteobacteria (class level); Pseudomonadales, Burkholderiales, and Enterobacterales (order level); Moraxellaceae, Comamonadaceae, and Enterobacteriaceae (family level); *Acinetobacter*, *Citrobacter*, and *Escherichia* (genera level), and *Escherichia coli* (species level). On the other hand, the dominant bacteria of tissue (LDA > 4) were Actinobacteria and Synergistetes (phyla), mainly Actinobacteria and Synergistia (class); Micrococcales and Oceanospirillales (order); Intrasporangiaceae, Alcanivoracaceae, and Synergistaceae (family); *Tetrasphaera* and *Alcanivorax* (genera), and *T. japonica*, and *A. hongdengensis* (species). Our results indicate that the dominant bacteria in IVF are different from those in tissue (LDA score histogram and the evolutionary branching plot are shown in Figures 4A,B).

### Correlation of Bacterial Communities and Clinical Parameters

**FIGURE 5:**
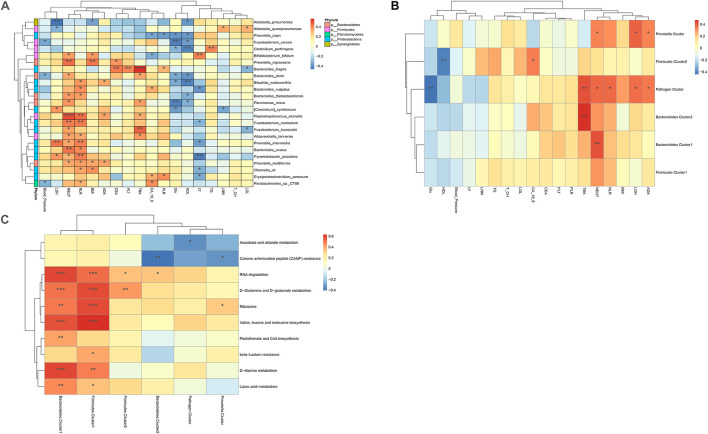
**(A)** Heatmaps of interactions between species and environmental factors. Environmental factors are on the X-axis, and species are on the Y-axis. **(B)** Heatmap of the relationship between clusters and environmental factors. The X-axis is the environmental factor, and the Y-axis is the cluster. **(C)** Heatmap of the relationship between metabolic pathways and clusters. The X-axis shows the clusters, and the Y-axis shows the metabolic paths. The R-value (rank correlation) and the *p*-value of the corrected error finding rate were calculated. The R values are shown in different colors in the figure. The legend on the right is the color code of the different R values. *p < 0.05, **p < 0.01, ***p < 0.001.

To study the influence of bacteria or bacterial clusters on parameters reflecting patient disease characteristics, we identified the associations between bacteria genera and the following confounding factors: body mass index (BMI), metabolic parameters, including total bile acid (TBA), glucose, and low-density lipoprotein (LDL)/high-density lipoprotein (HDL), inflammatory parameters, including neutrophils, and tumor markers such as CA-19-9 and CEA. Neutrophil count was positively correlated with *Bifidobacterium bifidum*, *Prevotella nigrescens*, *B. dorei*, *Bacteroides thetaiotaomicron*, *Parvimonas micra*, *Peptostreptococcus stomatis*, *Fusobacterium nucleatum*, *Fusobacterium hwasookii*, *Alloprevotella tannerae*, *Prevotella intermedia*, *Bacteroides ovatus*, *Pyramidobacter piscolens*, and *Prevotella multiformis* (*p* < 0.05). BMI index was positively correlated with *B. bifidum*, *P. nigrescens*, *P. multiformis*, and *B. ovatus* (*p* < 0.05); TBA was positively correlated with *B. fragilis/dorei*, *P. micra*, *P. stomatis*, *F. hwasookii*, and *A. tannerae* (*p* < 0.05); glucose (Glu) and HDL were found to negatively correlate with *P. copri*, *Fusobacterium varium*, *Clostridium perfringens*, *B. dorei*, *Bilophila wadsworthia*, *P. micra*, and *Clostridium symbiosum* (*p* < 0.05); and tumor marker CA-19-9 was found to be negatively correlated with *P. copri* and *B. bifidum*, but positively correlated with *B. vulgatus*, *Erysipelatoclostridium ramosum*, and *Parabacteroides_sp._CT06* (*p* < 0.05). CEA was positively correlated with *P. nigrescens* and *B. fragilis* (*p* < 0.05). Our results suggest that some intestinal bacteria could influence the metabolic and inflammatory parameters of CRC patients. Conversely, other bacteria had no significant influence (Figure 5A).

The correlation between bacterial clusters and patient characteristics was also analyzed. TBA was positively correlated with Bacteroidetes 1, Bacteroidetes 2, and Pathogen Cluster (*p* < 0.01). Neutrophil count was positively correlated with Bacteroidetes 1, Pathogen Cluster, and *Prevotella* Cluster (*p* < 0.05); Pathogen Cluster and *Prevotella* Cluster were also positively associated with the metabolic parameters LDL and ADA. Pathogen Cluster was negatively associated with Glu (*p* < 0.01), and Firmicutes Cluster 2 was negatively associated with HDL (*p* < 0.01). The correlation between bacteria and patient characteristics is depicted as a heatmap (Figure 5B).

We used the pheatmap tool to identify potential differences in the metabolic capability of each cluster between sample groups. Bacteroidetes 1 and Firmicutes 1 were the two clusters that had the most influence on signaling pathways, both of which had a close association with the signaling pathways connected with RNA degradation, D-glutamine, and D-glutamate metabolism, ribosome, valine, leucine and isoleucine biosynthesis, D-alanine metabolism, and lipoic acid metabolism. In addition, Bacteroidetes 1 was related to pantothenate and CoA biosynthesis, and Firmicutes 1 was related to beta-lactam resistance. On the contrary, Bacteroidetes 2 and *Prevotella* Clusters were negatively associated with cationic antimicrobial peptide (CAMP) resistance. Pathogen Cluster was negatively associated with ascorbate and alternate metabolism (Figure 5C).

### Identification of Operational Taxonomic Units That Are Associated With Antibiotic Resistance Genes

A total of 1,295 ARGs were detected in the gut microbiomes. We selectively studied the top 100 ARGs based on their abundance, and matched each resistance gene type to its corresponding antibiotic, then summarized the relative abundance of types resistant to the same antibiotic. The subtypes (>5) belong to the multidrug resistance types (27), followed by resistance to aminoglycosides (12), tetracycline (10), peptide antibiotics (8), dual drugs (6), and fluoroquinolone antibiotics (5). Our results showed that ARGs of multidrug resistance, tetracycline, and aminoglycosides were the dominant types in the human gut. Among all ARGs, VatI, MdtE, TetQ, ErmB, AAC(3)-IIa, MphA, MdtF, Sul1,TolC, PmrE, BaeR, PmrF, AcrE, CRP, MdtP, MdtM, bacA, MsbA, MdtB, and ErmF were the top 20 ARGs in both IVF and tissue. We then compared ARG richness between IVF and tissue samples based on the number of subtypes and total abundance for each sample. Procrustes analysis showed that ARG subtypes had significant associations with microbial species. In IVF, ARG-carrying bacteria mainly were *Bacteroides* (*fragilis/vulgatus*), *Citrobacter* (*freundii/complex_sp._CFNIH9/youngae*), *E. coli*, *K. pneumoniae*, *P. copri*, and *Tepidimonas fonticaldi*. While in the tissue, the most ARG-carrying bacteria were *B. vulgatus*, *Colwellia marinimaniae*, *P. antarcticus*, *E. coli*, *A. hongdengensis*, *B. thetaiotaomicron*, *P. piscolens*, and *T. japonica*. Our results indicate that the main ARG-carrying bacteria, i.e., *Bacteroides* and *E. coli* are similar between IVF and tissue.

**FIGURE 6:**
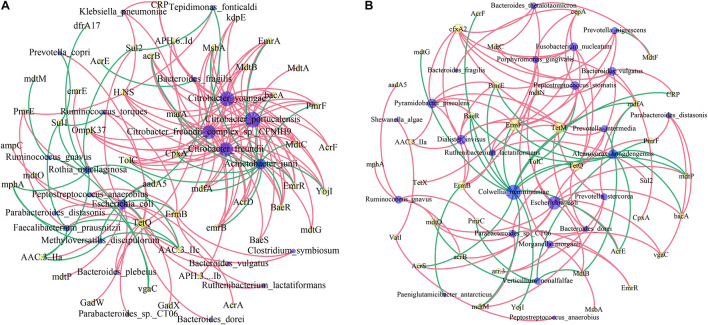
Network analysis of co-occurrence patterns among antibiotic resistance gene (ARG) subtypes and microbial taxa in IVF **(A)** and tissue **(B)**. The nodes are colored according to ARG types and species. A connection represents a strong (Spearman’s correlation coefficient *r* > 0.4) and significant (*p* < 0.05) correlation. The size of each node is proportional to the number of connections.

We then investigated the co-occurrence patterns between ARG types and microbial taxa using the network analysis approach. Pairs with Spearman r > 0.4 and *p* < 0.05 were used to stand for co-occurrence patterns. Bacterial genera were speculated as possible ARG-type hosts based on co-occurrence results. In IVF, a total of 69 nodes (23 species and 46 ARG subtypes) and 161 edges (subtype–species connections) were included in the co-occurrence network. The figure clearly shows that the leading bacterial hubs were *Citrobacter*, *Acinetobacter junii*, and *E. coli.* Three subtypes of *Citrobacter* (*portucalensis/youngae/freundii/freundii_complex_sp._CFNIH9*) were the hub node that connected with several ARGs. *A. junii* and *E. coli* were also hub nodes with a high degree of connectedness. *E. coli* co-occurred with ARG subtypes, including five multidrug resistance genes (e.g., mdtP, mdtO, GadW, GadX, AcrA), 3′-aminoglycoside [AAC(3)-IIa, AAC(3)-IIc, aadA5], 2 quinolone (EmrR, emrB), 1 Sul2, TetQ, and ampC. In tissue, a total of 62 nodes (24 species and 38 ARG subtypes) and 116 edges (subtype–species connections) were included. The leading hubs were *C. marinimaniae* that connected with seven multidrug resistance genes (CRP, AcrS, mdfA, mdtP, mdtM, AcrF, and AcrE), one dual resistance (BaeR), two peptide antibiotics (YojI, PmrE), and one of TetQ, ErmF, and mdtG. *E. coli* co-occurred with ARG subtypes, including eight multidrug resistance genes (AcrS, AcrE, acrB, vgaC, mdtN, mdtO, mdtP, and TolC), two peptide antibiotics (bacA and PmrF), and one MdtB. Other leading bacteria were *A. hongdengensis*, *Dialister invisus*, *P. piscolens*, and *B. vulgatus.* Except *for E. coli*, bacteria located in the hub were quite different between IVF and tissue, and the microbiota network showed a significantly different bacterial distribution (Figures 6A,B).

### Analysis of Microbial Community Function/Metabolic Pathway With Intestinal Lavage Fluid and Tissue

**FIGURE 7:**
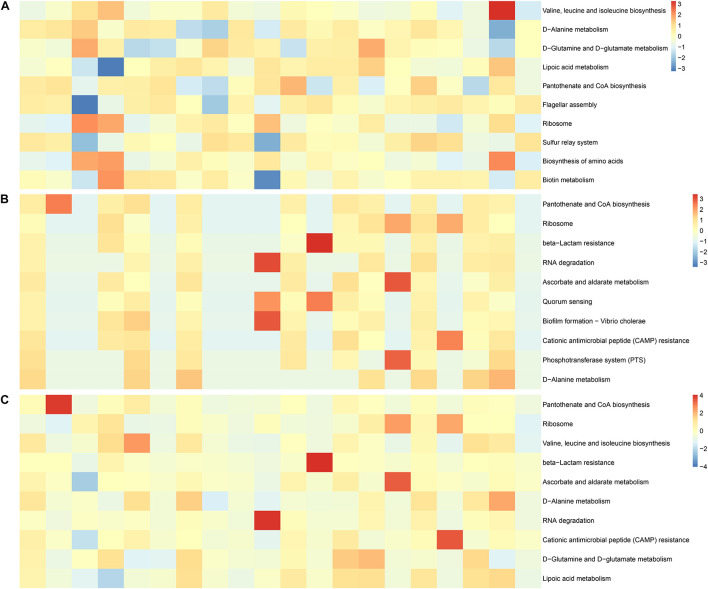
Functional features of the gut microbiome. **(A)** The 10 most abundant metagenomic pathways in IVF. **(B)** The 10 most abundant metagenomic pathways in tissue. **(C)** The 10 most abundant metagenomic pathways in the two groups. The heatmap is converted from standardized data and log2.

We analyzed the factors representing metabolic pathways by metagenomics, and the top 10 metabolic pathways are shown in Figure 7. After the conversion of standardized data and log2, they were ranked according to abundance. The main metabolic pathways were for valine, leucine, and isoleucine biosynthesis, D-glutamine and D-glutamate metabolism, lipoic acid metabolism, flagellar assembly, biosynthesis of amino acids, sulfur relay system, and biotin metabolism were abundant in the IVF, whereas beta-lactam resistance, RNA degradation, ascorbate and aldarate metabolism, quorum sensing, biofilm formation—*Vibrio cholerae*, CAMP resistance, and phosphotransferase system (PTS) were abundant in tissue. D-Alanine metabolism, ribosome, and pantothenate, and CoA biosynthesis were found in both IVF and tissue. Our results indicate that metabolic pathways detected in IVF or tissue samples were incomplete same (Figure 7).

## Discussion

The gut microbiome has emerged as a central player in CRC pathogenesis, and it has been shown to have multiple effects on tumor biology, such as the transformation process, tumor progression, and the response to anticancer therapies, including immunotherapy (Wong et al., 2017; Matson et al., 2018; Helmink et al., 2019). Some bacteria play a leading role in the occurrence and development of CRC. Previous studies have yielded inconsistent results. In one report, CRC tumor biopsy specimens were shown to harbor greater abundances of Fusobacteria and Actinobacteria, and their paired adjacent tissue counterparts harbor an elevated abundance of Firmicutes (Shah et al., 2018). However, in another study, a lower abundance of Bacteroidetes, Firmicutes, and Actinobacteria, and higher abundance of Proteobacteria and Fusobacteria were observed in tumor tissues (Wang et al., 2020). Compared with their tumor biopsy counterparts, fecal samples harbor a greater abundance of *Verrucomicrobia roseburia*, *Blautia*, *Bifidobacterium*, and fewer *Proteobacteria* and *Prevotella*, suggesting that the bacterial microbiota varies in different types of samples or anatomical positions. Bacteria existing as the abundant taxon of CRC tumor tissues are protagonists of tumor development, due to their close interactions with epithelial and immune cells, and their presence correlates with increased risks of CRC. Up to now, *F. nucleatum*, *E. coli*, enterotoxigenic *B. fragilis*, *S. gallolyticus*, and *Klebsiella*, among others, have been successively discovered to act as driver bacteria for CRC carcinogenesis (Swidsinski et al., 1998; Tjalsma et al., 2012; Wang et al., 2012, 2020; Antonic et al., 2013; Sears and Garrett, 2014; Tahara et al., 2014). Therefore, the microbiota of tumor tissue may provide extremely valuable information in identifying the tipping point in malignant transformation, detecting stages of carcinogenesis, and evaluating the potential prognosis of CRC (Shah et al., 2018). However, due to the aforementioned limitations in tissue samples, plus the considerable variation in composition and abundance of the gut microbiota across anatomic sublocations in the colorectum (Donaldson et al., 2016; Purcell et al., 2017), there are still uncertainties and inconsistent results when detected from tissue specimens (Flemer et al., 2017; Shah et al., 2018; Wang et al., 2020). Furthermore, the CRC-associated microbiome is dynamic, with changes occurring during CRC progression. Interindividual microbial community heterogeneity of the human gut is influenced by spatial distribution, microheterogeneity, and host genetics, which has posed a long-standing challenge when investigating microbial signatures implicated in CRC tumorigenesis (Eckburg et al., 2005; Hong et al., 2011; Zhang et al., 2014; Thomas et al., 2016; Pan et al., 2020). Differences in analysis, methodology (e.g., phylogeny, culturing, and metagenomics), and sample size can also lead to markedly different findings (Walker et al., 2014). Uncovering specific microbiota structures with suitable specimens will benefit our understanding of how microbial dysbiosis impacts the microenvironment of the colon.

The biofilm-like architecture of the mucosal microbiota, in close contact with the underlying gut epithelium, facilitates nutrient exchange and induction of host innate immunity (Sonnenburg et al., 2004). The stability of intestinal microbiota is achieved in part through the ability of these microbes to attach to the mucosa (Harrell et al., 2012). Thus, mucosal adherent bacteria are likely to play a more direct role in the pathogenesis of CRC than luminal bacteria (Keku et al., 2015). Currently, the mucosa-associated gut microbial richness and biodiversity shifts associated with CRC progression have remained largely unexamined (Flemer et al., 2017). IVF is obtained from patients preparing for laparoscopic colorectal resection. Some species of mucosa-associated microbiota (either on the surface or in cavities) may be obtained after a few whole intestinal rinses. Thus, IVF is enriched in mucosal microbes from the entire colonic environment and includes areas that tissue biopsy cannot reach (Shen et al., 2020). A previous study reported that microbiota of paired mucosal and fecal samples from individuals with CRC differed significantly (Flemer et al., 2017; Russo et al., 2017; Shen et al., 2020). Due to intestinal flora being susceptible to food and antibiotics, fecal bacteria fluctuate greatly and likely do not accurately reflect the mucosal microbiota composition. Given the known limitations of tissue sampling, we set out to investigate whether IVF was an alternative approach. Our results show that a greater number of bacterial species is detected in IVF than that in tumor tissue (2,757 species vs. 197 species). Similarly, higher bacterial diversity is detected in IVF samples than in tissue, particularly pertaining to species richness. Bowel preparation may alter microbial alpha and beta diversity (Harrell et al., 2012; O’brien et al., 2013). On the other hand, it is likely that luminal bacteria have a more complex community structure (Keku et al., 2015). Beta diversity revealed that distinguishing cluster of bacteria formed between tissue and IVF groups. When the differences at each bacterial level between the two specimen groups were further studied, we found that Proteobacteria and Verrucomicrobia were higher in IVF, while Bacteroidetes and Actinobacteria were higher in tumor tissue. Verrucomicrobia and fewer Proteobacteria mainly existed in the fecal of CRC, while abundant Fusobacteria and Actinobacteria existed in tumor tissue (Shah et al., 2018). In our study, Fusobacteria did not significantly differ in both tissue and IVF, suggesting that bacterial composition in IVF could reflect either fecal or tissue microbiota. *Fusobacteria* is a key bacterium in CRC carcinogenesis, which is enriched in CRC tissues (Tahara et al., 2014). Some bacteria, including *E. coli*, *K. pneumoniae*, *B. fragilis*, and *B. vulgatus*, presented in large quantities in both IVF and tissue, suggesting that IVF could provide a comparative assessment of microbial diversity without the limitations associated with biopsy collection (Watt et al., 2016).

Heterogeneity of microbiota is particularly evident when it was studied at the level of CAGs rather than at the level of individual organisms or taxa. Microbial clusters were considered a higher-level structure of the CRC-associated microbiota, reflecting gut microflora better than a particular bacterium. However, inconsistent results have also been observed in various studies (Kostic et al., 2012; Zeller et al., 2014; Flemer et al., 2017). A previous study showed that Firmicutes Cluster 1 and Bacteroidetes Cluster 1 were significantly less abundant in the microbiota of individuals with CRC. In contrast, other studies have shown that bacteria belonging to the Firmicutes and Bacteroidetes phyla are the most abundant species in CRC (Gao et al., 2015; Nejman et al., 2020), and that Firmicutes Cluster 2, *Prevotella* Cluster, Pathogen Cluster, and Bacteroidetes Cluster 2 are more abundant in CRC biopsy microbiota (Flemer et al., 2017). From a functional point of view, the microbiota of individuals with high abundances of the *Prevotella* Cluster and Pathogen Cluster might influence the development of CRC through modulating the expression of immunoinflammatory response genes (i.e., CXCL1), which has been shown to increase the survival of cancerous cells and promote angiogenesis in CRC(Wang et al., 2006; Acharyya et al., 2012; Flemer et al., 2017). In our study, there was no significant difference between Firmicutes Cluster 2, Bacteroidetes 2, Pathogen Cluster, and *Prevotella* Cluster in the IVF group and those in the tissue group, demonstrating that microbiomes in IVF could reflect those in the tissue. Pathogen Cluster is associated with increased TH17 response and may be associated with a poor prognosis for CRC (Flemer et al., 2017). However, the Pathogen Cluster is in very low abundance in fecal microbiota (Flemer et al., 2017), making it difficult to detect in feces. Thus, detecting it with IVF may provide more valuable information mirroring its content in tissues. The abundance of Firmicutes Cluster 2 and Bacteroidetes Cluster 2 is also correlated with a mucosal gene-expression profile more resembling that of a healthy mucosa (Flemer et al., 2017).

The relative abundance of these CAGs differs by tumor location. For example, Bacteroidetes Cluster 2 and the Pathogen Cluster are more abundant in distal cancers, whereas the *Prevotella* Cluster and Firmicutes Cluster 2 are only significantly more abundant in individuals with proximal cancers (Flemer et al., 2017). As IVF enriches the mucosal microbes from the entire colonic environment, the differences in spatial distribution are alleviated. From this point, bacterial microbes in tissue more accurately reflect the relationship between bacterial flora and cancer. Therefore, selecting suitable specimens according to the purpose of the study is essential for a better understanding of the host–microbial relationships in health and disease.

Presently, a full understanding of how the bacteria microbiome impacts host metabolism is still lacking. *Bacteroides* species are closely associated with an increased risk of CRC because of their ability to convert bile to fecapentaenes, considered carcinogenic or mutagenic metabolites (Moore and Moore, 1995). In our study, *Bifidobacterium*, *Prevotella*, and *Bacteroides* were positively correlated with BMI index. Bacteria, including *Bacteroides*, *Parvimonas*, *Peptostreptococcus*, *Fusobacterium*, and *Alloprevotella tannerae*, as well as Bacteroidetes 1, Bacteroidetes 2, and Pathogen Cluster, influence the TBA. Glu and HDL are negatively correlated with Firmicutes Cluster 2, *Prevotella copri*, *Fusobacterium, Clostridium*, and *Bacteroides*. The Pathogen Cluster and *Prevotella* Cluster are also positively associated with the metabolic parameters LDL and ADA. Pathogen Cluster is negatively associated with Glu. These results indicate the effects of the intestinal microbiome on metabolism.

Antibiotic administration results in a fundamentally altered gut microbiome, and the stable microbiome has remarkable plasticity to routine perturbation of antibiotics, but the response to each antibiotic differs based on the microbiome composition (Gibson et al., 2016; Schwartz et al., 2020). The gut microbiome can act as a reservoir for antibiotic-resistant bacteria and their associated genes (ARGs) (Hu et al., 2013). ARGs transmit between species within the gut microbiome, including potential pathogens. Therefore, understanding how the resistome changes in parallel with the microbiome is vitally important in CRC (Stalder et al., 2019). Nowadays, only a few studies have characterized the effects of particular antibiotic regimens on the gut ecosystems of individuals with respect to the associated resistome (Palleja et al., 2018), which may be because data for antibiotic exposure on gut microbiota are quite hard to obtain. To assess the resistomes in IVF and tissue samples, we used a measure for the antibiotic resistance potential of a microbial community based on the abundance of its resistance genes relative to its species composition. We then matched each ARG type to its corresponding antibiotic and summarized the relative abundance of types resistant to the same antibiotic. Our results reveal that the common ARGs, detected in both IVF and tissue, confer multidrug resistance, as well as resistance to aminoglycosides, beta-lactams, and tetracycline antibiotics. A previous study showed that tetracycline, aminoglycoside, beta-lactam, MLS, vancomycin, and multidrug resistance genes are the dominant types in the human gut (Qiu et al., 2020). The human microbiome may constitute a mobilizable reservoir of ARGs, which are accessible to pathogenic bacteria for acquiring antibiotic resistance. However, direct experimental proof of *in vivo* transfer of antibiotic resistance genes within the human microbiome remains to be shown (Sommer et al., 2009). Characterizing resistome distribution and its relationship with the gut microbiota can help elucidate the effects of antibiotic use in CRC pathogenesis and manage antibiotics at the clinical practice.

The gut microbiota network shows different bacterial hubs in tissue groups compared with IVF microbiota. The leading bacterial hubs in IVF are *Citrobacter*, *A. junii*, and *E. coli*, which relate to many ARGs. While in the tissue, the leading hubs were *Colwellia*, *E. coli*, and *B. vulgatus. E. coli* is the only bacterium located in the central hub of IVF and tissue and contains similar ARGs in the two sample groups. Most ARGs in the human gut are those resistant to widely used antibiotics, and antibiotic therapy leads to personalized resistome diversification and individual-specific strain level selection in the gut microbiota (Li et al., 2019). Analysis of ARG distribution in the population provides an important indicator for public health policies (Qiu et al., 2020).

The characterization of the tumor microbiome has remained challenging because of its low biomass, methodology of detection, and sample size (Walker et al., 2014; Nejman et al., 2020). Evaluating on-tumor versus off-tumor microbial communities, and mucosal versus fecal taxonomic disparities in the context of CRC, have been hindered by the limited number of studies that have examined differences in both the mucosal (both tumor and tumor-adjacent tissue) and fecal microbiota within the same CRC cases (Chen et al., 2012; Weir et al., 2013; Mira-Pascual et al., 2015; Flemer et al., 2017). Fecal sample collection still offers the benefit of participants not needing to have a colonoscopy to provide samples. Therefore, attracting a large sample size is not an issue (Watt et al., 2016). Even though fecal microbiota partially reflects the microbiota at the mucus layer, differences between fecal and tissue microbiota are still evident. The bacterial clusters in IVF may provide greater insight into mucosal-specific colonic neoplasia due to their close interactions with epithelial and immune cells (Hong et al., 2019). As there is no unanimous conclusion in the advantages of different types of specimens, accurate comparisons between sample types in large sample sizes are needed, and a combinatorial approach may enable a sensitive and accurate evaluation of the gut microbiota.

The main limitation of this study is the small sample size. Nowadays, patients do not need intestinal lavage before doing a colonoscopy in general clinical practice, then the paired sample is extremely difficult to obtain. The results would be more valuable if microbiome detects in various anatomical locations (rectum, proximal colon, distal colon) as well as different TNM stages.

## Data Availability Statement

The datasets presented in this study can be found in online repositories. The names of the repository/repositories and accession number(s) can be found below: https://www.ncbi.nlm.nih.gov/, PRJNA754518.

## Ethics Statement

The studies involving human participants were reviewed and approved by The Shantou University Medical College Institutional Ethics Board. The patients/participants provided their written informed consent to participate in this study. Written informed consent was obtained from the individual(s) for the publication of any potentially identifiable images or data included in this article.

## Author Contributions

XJ, YY, YC, and FY designed and drafted the manuscript. XJ revised the final manuscript and had full access to all of the data in the study and took responsibility for the integrity of the data and the accuracy of the data analysis. YC collected the samples. YY finished the experiment. MS and SN summarized the clinical data. YY, MZ, and QX processed the statistical data. All authors contributed to the data analysis, drafting or revising the article, had agreed on the journal to which the article will be submitted, gave final approval of the version to be published, and agreed to be accountable for all aspects of the work.

## Conflict of Interest

The authors declare that the research was conducted in the absence of any commercial or financial relationships that could be construed as a potential conflict of interest.

## Publisher’s Note

All claims expressed in this article are solely those of the authors and do not necessarily represent those of their affiliated organizations, or those of the publisher, the editors and the reviewers. Any product that may be evaluated in this article, or claim that may be made by its manufacturer, is not guaranteed or endorsed by the publisher.
